# Nano-Brick Wall Architectures Account for Super Oxygen Barrier PET Film by Quadlayer Assembly of Polyelectrolytes and α-ZrP Nanoplatelets

**DOI:** 10.3390/polym10101082

**Published:** 2018-09-29

**Authors:** Dongmei Han, Yiqing Luo, Qing Ju, Xujing Xiao, Min Xiao, Naiyu Xiao, Shou Chen, Xiaohua Peng, Shuanjin Wang, Yuezhong Meng

**Affiliations:** 1 The Key Laboratory of Low-carbon Chemistry & Energy Conservation of Guangdong Province/State Key Laboratory of Optoelectronic Materials and Technologies, Sun Yat-Sen University, Guangzhou 510275, China; handongm@mail.sysu.edu.cn (D.H.); luoyq6@mail2.sysu.edu.cn (Y.L.); juqing@mail2.sysu.edu.cn (Q.J.); xiaoxj6@mail2.sysu.edu.cn (X.X.); stsxm@mail.sysu.edu.cn (M.X.); xiaony81@163.com (N.X.); 2 School of Chemical Engineering and Technology, Sun Yat-Sen University, Guangzhou 510275, China; 3 Shenzhen Beauty Star Co., Ltd., Shenzhen 518112, China; chens@beautystar.cn (S.C.); alice@beautystar.cn (X.P.)

**Keywords:** nanobrick, electrostatic interaction, oxygen barrier, packaging applications

## Abstract

Nanobrick wall hybrid coating with super oxygen barrier properties were fabricated on polyethylene terephthalate (PET) film using a quadlayer (QL) assembly of polyelectrolytes and nanoplateles. A quadlayer assembly consists of three repeat units of polyacrylic acid (PAA), poly (dimethyl diallyl ammonium chloride) (PDDA) and layered α-zirconium phosphate (α-ZrP). PDDA with positive charges can assemble alternatively with both α-ZrP and PAA with negative charges to form nanobrick wall architectures on the surface of PET film via the electrostatic interaction. The lamellar structure of α-ZrP platelets and the dense QL assembly coating can greatly reduce the oxygen transmission rate (OTR) of PET film. Compared to pristine PET film, the OTR of PET (QL)_19_ is reduced from 57 to 0.87 cc/m^2^/day. Moreover, even with 19 QLs coating, PET (QL)_19_ composite film is still with an optical transparency higher than 90% and a haze lower than 10%. Therefore, the transparent PET (QL)_n_ composite films with super oxygen barrier properties show great potential application in food packaging and flexible electronic packaging.

## 1. Introduction

Barrier materials have been widely used in the fields of food preservation, pharmaceuticals and electronic packaging to protect them from detrimental effect of moisture and oxygen in the environment [1]. As traditional barrier materials, metal and glass materials offer excellent barrier properties to gases, but they are not suitable for the flexible and lightweight packaging [2]. As replacements for metal and glass materials, polymer materials are normally inexpensive, transparent, lightweight and easily processable, but their barrier performances are not good enough because of the large free volume between macromolecular chains. The free volume can provide convenience for gas permeation [3,4,5]. Considerable efforts have been devoted to improve the barrier properties of polymeric barrier materials, such as (1) thin and transparent vacuum deposited coatings, (2) new barrier polymers as discrete layers, (3) blends of barrier polymers and standard polymers, (4) organic/inorganic barrier coatings, and (5) nanocomposite materials [1]. However, barrier films with super gas barrier performance, high transparency and good flexibility are still challenging. To achieve super gas barrier requirements need for packing, different hybrid consisting of polymeric matrices and two-dimensional (2D) inorganic platelets have been developed by layer-by-layer (LbL) method [6,7,8,9].

LbL assembly was first reported by Decker [10] and has been widely used as a simple and versatile thin-coating fabrication method. Multilayer coating can be constructed by alternating deposition of different species with negative and positive charges. Resultant multilayer coatings can increase the gas barrier properties of polymer substrate [11]. In the case of 2-dimentional inorganic nanoplatelets with negative charges, sodium montmorillonite (MMT) [8], Laponite (LATP) [9] and grapheme oxide (GO) [12] have been used to construct LbL thin coatings with positively charged polyelectrolytes. The thin LbL coatings exhibit a nanobrick wall structure with high 2D nanoplatelet alignment. This structure induces a long diffusion length of oxygen and water vapor molecules. A variety of LbL coating, such as polyallylamine (PAAm)/polyacrylic acid (PAA)/MMT [13,14], polyethylenimine (PEI)/MMT [15,16,17], PEI/vermiculite (VMT) [18] and PEI/graphene oxide [19] have been reported to increase the barrier performance of polymer based barrier packaging materials.

Poly(ethylene terephthalate) (PET) has been widely studied and used in the fields of food and pharmaceuticals packaging because of its remarkable advantages, such as high transperency, high mechanical strength, dimensional stability, low cost, and relatively low permeabilities to both oxygen and water vapor [20]. In order to get PET composite film with super gas barrier properties, several methods have been reported, such as: (1) Melt blending with higher barrier polyamides [21] or nanoplates [22], (2) Deposition of a thin SiO_x_ or metal oxide onto a surface by vacuum vapor deposition process [23,24], (3) LbL self-assembly of polyelectrolytes or inorganic nanoplatelets with the opposite charges. LbL method has been extensively studied and proved to be a low cost and effective way to improve the barrier properties of PET.

Negatively charged 2D inorganic nanoplatelets, such as MMT, GO, and zirconium phosphate (ZrP), have been reported to construct bilayer (BL) assembly films onto the PET film with positively charged polyelectrolytes [8,9,10,11,12,13,25]. LbL transparent thin films containing inorganic components have been investigated for their high strength, anti-flammability, and oxygen barrier properties [26,27,28]. The oxygen barrier properties can be dramatically improved by switching BL to quadlayer (QL) assembly, which consists three layers of oppositely charged polyelectrolytes between each nanoplatelets layer [29] and made with cationic polyethyleneimine (PEI) and anionic MMT and PAA. One deposition sequence of PEI/PAA/PEI/MMT is referred to as a quadlayer (QL). Greater clay spacing in the QL film than in the BL film are responsible for more room for gas molecule wiggling, leading to a tortuous diffusion length and improving the gas barrier property.

As an layered metal phosphate hydrate (LMPs), the layered α-zirconium phosphate (α-ZrP) can be easily synthesized and exfoliated into negatively charged monolayer nanoplatelets by using the tetrabutylammonium hydroxide (TBAOH) as the exfoliated agent [30,31,32]. α-ZrP nanoplates have been widely used in corrosion protection [33] and flame retardation with methods of melt blending [34], solution blending [35,36] and in-situ polymerization [37]. Utilizing the strong negative surface charges, α-ZrP nanoplates can be used in the electrostatic LbL assembly to fabricate a nanobrick wall architecture coating. On the other hand, it was found that the spacing and orientation of nanoparticles effect the nanostructure of the composites and thus the permeability in a packaging matrix [38,39].

In one of our previous work, a novel LbL composite membrane, Nafion-[PDDA/ZrP]_n_ with nacre-like structures has been reported to effectively suppress the vanadium permeation in vanadium redox flow battery [40]. The results showed that the lamellar structure of α-ZrP nanoplates generate more tortuous diffusion paths of ions and serves as barriers to prevent vanadium ions migrating though the membrane. Moreover, poly(acrylic acid) (PAA) exhibits high oxygen barrier properties and has been used improve the oxygen barrier properties of polymer matrix by coating method. But the hydroscopicity of PAA limits its application. We have reported a covalent layer-by-layer assembly method to fabricate a transparent and super-gas-barrier PET film by PAA based coating [41], in which the thick of coating film was difficult to control.

These previous works encouraged us to think how to fabricate a nanobrick wall architectures from α-ZrP nanoplates and PAA with the same negative charges for super gas barrier. Inspired by the QL assembly strategy reported by Grunlan et al. [29], we propose here a transparent and super oxygen barrier PET composite film with nanobrick wall architectures by a QL assembly, where cationic PDDA assembles with anionic PAA and α-ZrP nanoplatelets to fabricate a quadlayer of PAA/PDDA/ZrP/PDDA. The superior oxygen barrier property of QL assembly PET film were confirmed by oxygen transmission rate (OTR) measurement. The OTR values exponentially reduce with the number of QLs and achieve to only 1.5% of the pristine PET film after deposition 19 QLs, finally to undetectable oxygen transmission rate (OTR < 0.005 cm^3^/(m^2^ day atm)). The morphologies of QL films were investigated by SEM and AFM techniques. The improvement in the oxygen barrier properties originated from the alignment of lamellar α-ZrP nanoplatelets and the ion-crosslinking between cationic PDDA and anionic PAA andα-ZrP nanoplatelets, which created a tightly packed nanobrick wall microstructure, with PAA and PDDA as mortar, and α-ZrP nanoplatelets as bricks. The transparent and super oxygen barrier films presented in this work have great potential application in packaging fields.

## 2. Experimental Section

### 2.1. Materials

Zirconium oxychloride (ZrOCl_2_·8H_2_O, 98%, Aladdin, Shanghai, China), phosphoric acid (H_3_PO_4_, 85%, Aladdin, Shanghai, China), tetrabutylammonium hydroxide (TBAOH, 10 wt.%, TCI Shanghai, China), hexadecyl trimethyl ammonium bromide (CTAB, 99%, Aladdin, Shanghai, China), poly dimethyl diallyl ammonium chloride (PDDA, M_w_ = 100,000–200,000, 20 wt.% in water, Aladdin, Shanghai, China), polyacrylic acid (PAA, M_w_ = 100,000, 99.7%, Aladdin) were used as received. Ultrapure Milli-Q water (18.2 MΩ) was used for preparing all aqueous deposition solutions. Both PDDA and PAA solutions for deposition were with the concentration of 0.2 wt.%.

### 2.2. Substrates

Poly(ethylene terephthalate) (PET) film with a thickness of 25 μm (Chaozhou Xinde Packaging Materials Co., Ltd., Chaozhou, China) was used as the substrate for QL deposition and OTR testing. Prior to deposition, PET film were rinsed with deionized (DI) water and ethanol. In order to improve the adhesion of the first electrolyte layer, the dried and cleaned PET films were treated by corona treatment, and then with 0.2 wt.% CTAB for surface treatment. Silicon wafers were cleaned with piranha solution for 30 min and then rinsed with DI water, acetone several times, finally dried with filtered air prior to LbL depositon. Silicon wafer was used for AFM and SEM characterization.

### 2.3. Synthesis and Exfoliation of α-ZrP

α-ZrP with high crystalline was prepared according to the procedures described previously with some modifications [42,43]. 6.0 g of ZrOCl_2_·8H_2_O was dissolved in 10 mL DI water and rapidly mixed with 30 mL of 6M H_3_PO_4_ by vigorous stirring for 5 min. The result white suspension was transferred into a sealed Telfon-lined stainless autoclave and kept at 200 °C for 24 h. Then the white solid was washed with DI water by centrifuging until the pH of the supernatant reached 5–6. After dried in a vacuum oven at 60 °C for 12 h, the final α-ZrP was obtained and was exfoliated by TBAOH with ultrasound stirring at 0 °C for 3 h. The concentration of α-ZrP was controlled at 1 wt.% and the ultrasonication assistant was adopted to make the dispersed α-ZrP exfoliated as nanoplatelets by TBAOH in a short time. The 1 wt.% exfoliatedα-ZrP nanoplatelets dispersion was diluted to 0.2 wt.% with DI water for deposition.

### 2.4. Layer-by-Layer Deposition

**Figure 1:**
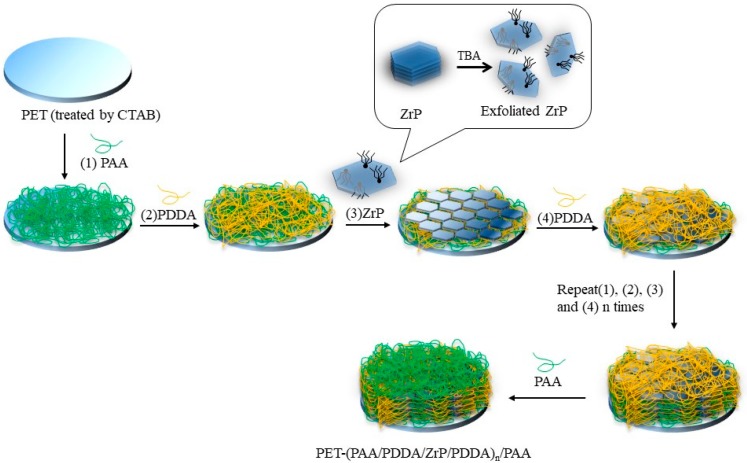
Schematic of the layer-by-layer (LbL) assembly for polyacrylic acid (PAA)/ poly (dimethyl diallyl ammonium chloride) (PDDA)/zirconium phosphate (ZrP)/PDDA quadlayers. PET: polyethylene terephthalate; TBA: tetrabutylammonium; CTAB: hexadecyl trimethyl ammonium bromide.

The overall LbL deposition process for QL is illustrated in Figure 1. The LbL assembly of QL multilayers was carried out by alternately immersing the substrates in the aqueous solutions of PAA, PDDA, α-ZrP and final PDDA for each 5 min at room temperature. After each deposition step, the film was rinsed with DI water to remove the weakly absorbed PAA, PDDA and α-ZrP nanoplatelets. One deposition sequence of PAA/PDDA/α-ZrP/PDDA is denoted as a QL. The PET (QL)_n_ films were fabricated by alternate deposition of QL for *n* cycles. The resulting composite film was denoted as PET (QL)_n_ and finally rinsed with DI water and dried with filtered air. All deposition processes were performed using a home-built dip coater. QL multilayers were also deposited onto silicon wafer by a same fabrication process.

### 2.5. Film Characterization

X-ray diffraction (XRD) patterns were recorded by Rigaku D/Max-IIIA X-ray diffractometer (Rigaku Corporation, Tokyo, Japan) using a nickel-filtered Cu-K_α_, at 40 kV and at a scan rate of 1°/min. Fourier transform infrared (ATR-FTIR) spectra were recorded by a PerkinElmer Spectrum 100 FTIR spectrometer over the wavenumber range of 4000–400 cm^−1^ to confirm the successful preparation of ZrP nanoplates. The sizes and distributions of the ZrP nanoplatelets were characterized by particle size analyzer (Elite Sizer, Brookhaven Instruments Corporation, Austin, TX, USA) which based on dynamic laser scattering (DLS).

The static state contact angle (θ) of PET and PET treated by CTAB were measured by SL200B contact angle measurement. Contact angle is an important indicator of wettability, classifying materials by hydrophobicity (θ > 90°) and hydrophilicity (θ < 90°). Typically, it is called spreading when θ does not exist or θ = 0°. Film thickness was measured (on silicon wafers) by the Ambios XP-1 step profiler (Ambios Technology Inc., Milpitas, CA, USA) under the optimal technological conditions. In order to obtain the thickness of QLs, half of silicon wafer was protected by a tape before deposition and then the tape was removed after deposition. The difference in height between the part with deposition and the part without deposition was measured, which is stand for the thickness of QLs.

The surface roughness and morphology of the composite films were measured, in air, by CSPM 5500 Atomic Force Microscopy (AFM, Benyuan Inc., Guangzhou, China) operating in the tapping mode. SEM images of α-ZrP nanoplatelets and the cross-section of the deposited QLs were recorded on a scanning electron microscopy (SEM) (Quanta 400, FEI, Hillsboro, OR, USA) operated at 15 kV. To investigate the morphology of QLs, PET(QL)_n_ was cut out of the center of the silicon wafer with QLs by a diamond blade and glued on cross-section holders. All specimens were coated with a thin layer of gold before SEM examination.

### 2.6. Gas Permeability

The oxygen transmission rates (OTRs) were measured by Y202D oxygen permeation analyzer (GBPI Packing Test Instruments Co. Ltd., Guangzhou, China). At a constant temperature of 23 °C under dry conditions according to ASTM D398-05 standard. And a WVTR analyzer (PERMATRAN-W Model 3/61, Mocon. Inc., Minneapolis, MN, USA) was used to measure the water vapor transmission rate (WVTR) at a constant temperature of 23 °C under 85% RH conditions with “Infrared detection sensor methods” (ISO 15106-2). The oxygen permeability coefficient (OP) and water vapor permeability coefficient (WVP) were calculated to evaluate the oxygen and water vapor barrier performance from film thickness, OTR (or WVTR) [44].

### 2.7. Hazes and Transmittances

The hazes and transmittances of PET composite films were measured using a haze and transmittance tester (Model: WGT-S, Shenguang Instrument Co. Ltd., Shanghai, China) at a constant temperature of 23 °C under 50% RH conditions according to GB 2410-2008 standard. The light transmittance (*T*_t_) and the haze (*H*) are calculated by the following Equations (1) and (2):

(1) $$T_{t}=\frac{T_{2}}{T_{1}}\times 100\%$$

(2) $$H=\left(\frac{T_{4}}{T_{2}}-\frac{T_{3}}{T_{1}}\right)\times 100\%$$

where *T*_1_ and *T*_3_ represent the incident optical luminous flux and the penetrated luminous flux respectively, and *T*_2_ and *T*_4_ are the scattering light flux of the instrument and the total scattering light flux of the instrument and sample, respectively.

## 3. Results and Discussion

### 3.1. Exfoliation of α-ZrP

**Figure 2:**
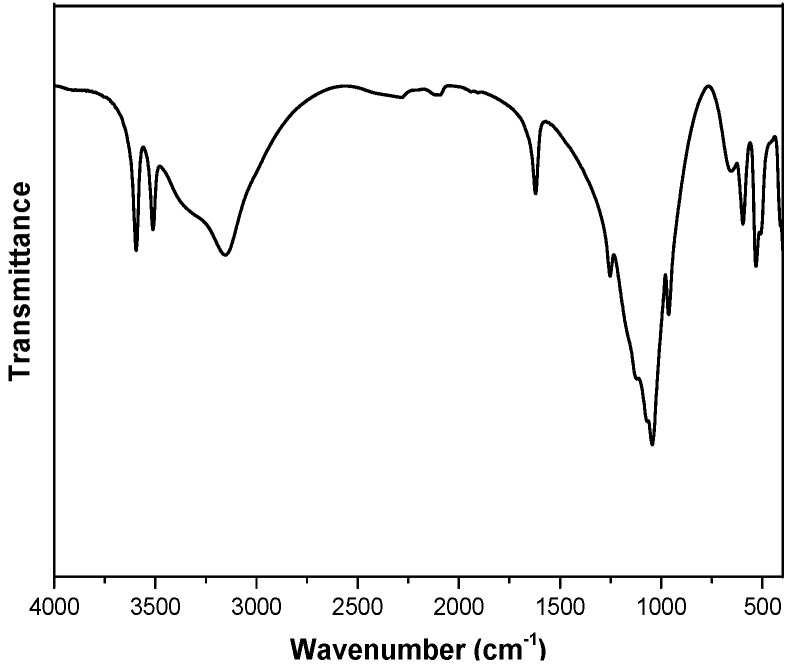
Fourier transform infrared (FTIR) spectrum of ZrP.

**Figure 3:**
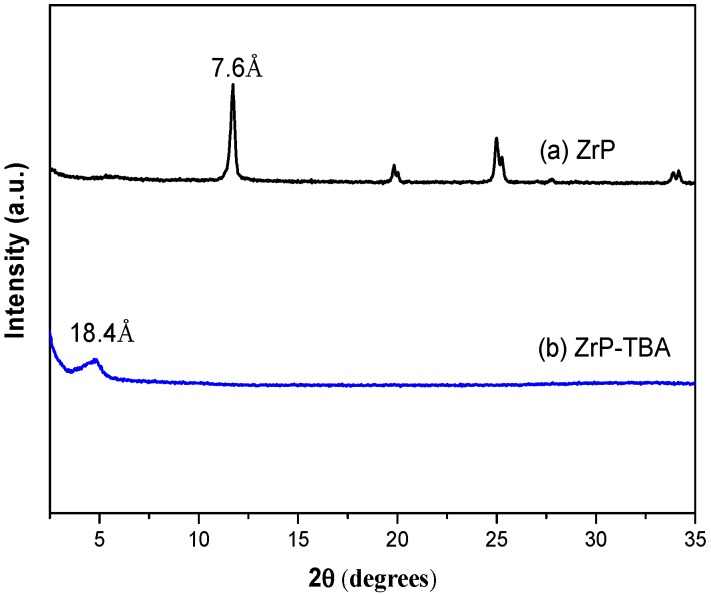
X-ray diffraction (XRD) pattern of (**a**) ZrP and (**b**)exfoliated ZrP nano platelets.

The FTIR spectrum of α-ZrP solid is presented in Figure 2. The bands at 3595 and 3512 cm^−1^ are attributed to the presence of interlayer water in α-ZrP. The stretching and bending vibration of -OH from lamellar surface is at 3165 and 1620 cm^−1^ respectively. The in-plane and out-of-plane vibrations of P-OH in PO_4_ groups are at 970 and 1251 cm^−1^ [45,46]. The bands at 1047 and 1121 cm^−1^ are attribute to the stretching vibration of O-P-O in PO_4_ groups. While the stretching vibrations of Zr-O are observed at 534 and 593 cm^−1^ [47]. The XRD patterns of α-ZrP and the exfoliated α-ZrP nanosheets are shown in Figure 3. α-ZrP was tested in a powder form, while the exfoliated α-ZrP was dried on a silicon wafer for XRD recording. As shown in Figure 3, the strong peak at 11.7° in the XRD pattern of crystalline α-ZrP indicates that the lamellas distance is 7.6 Å. The small peak at 4.8° in XRD pattern of the exfoliated α-ZrP is resulted from the less ordered restacking of the exfoliated α-ZrP nanoplatelets [48,49,50]. After exfoliation, the α-ZrP/TBAOH dispersion becomes more transparent and homogeneous than the pristine ZrP dispersion. The colloidal solution with exfoliated α-ZrP nanoplatelets has a “Tyndall effect”, which confirms the exfoliation of α-ZrP by TBAOH.

**Figure 4:**
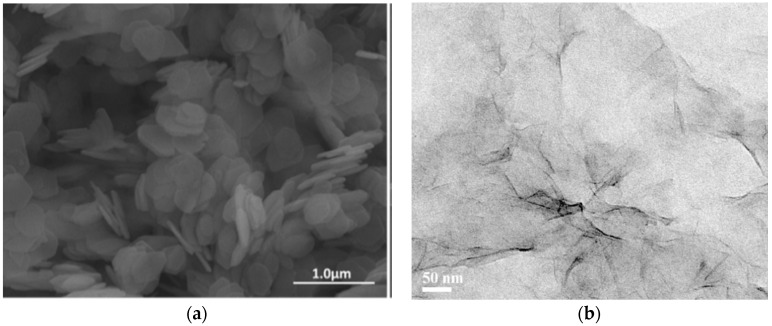

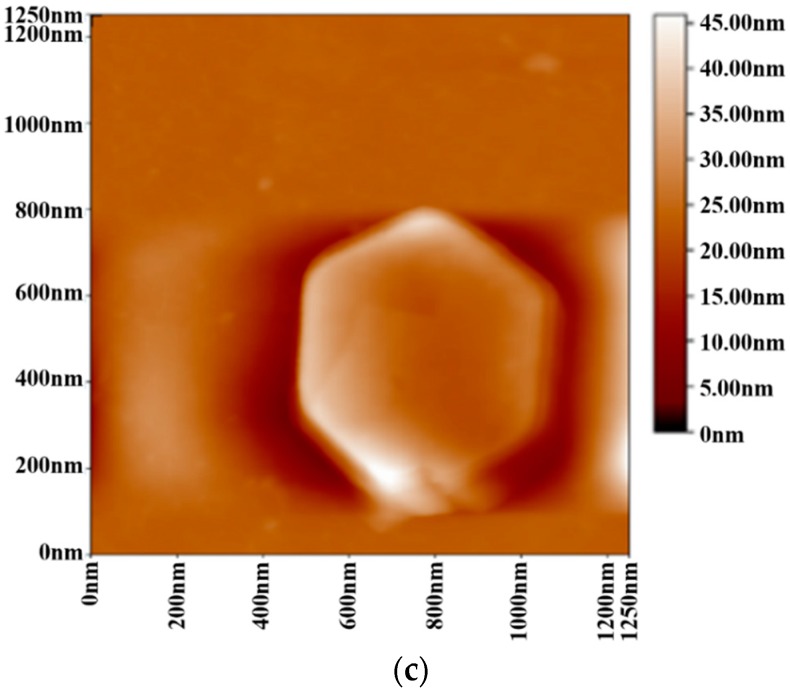
(**a**) Scanning electron microscopy (SEM) image of α-ZrP; (**b**) Transmission electron microscopy (TEM) image of exfoliated α-ZrP nanoplatelets; (**c**) Atomic Force Microscopy (AFM) image of exfoliated α-ZrP nanoplatelets.

**Figure 5:**
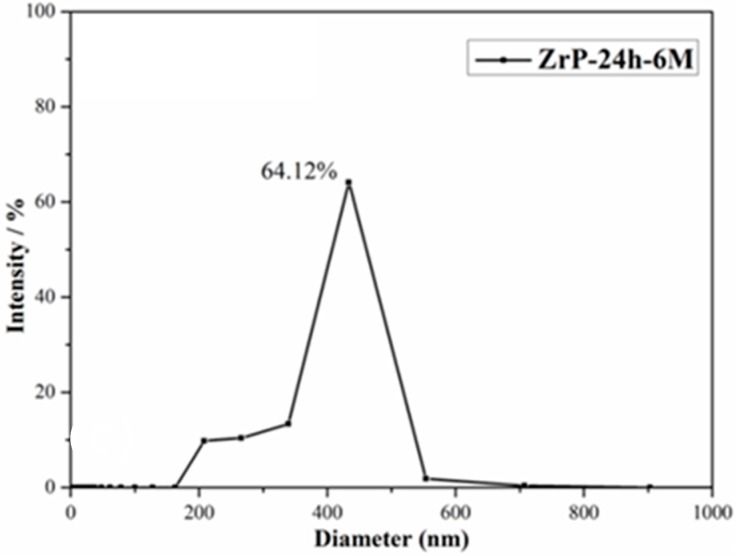
Particle size distribution of ZrP nanoplates.

SEM, TEM and AFM techniques are used to observe the morphologies of α-ZrP. Figure 4a shows the SEM image of α-ZrP and the regular hexagonal structure demonstrates the high crystalline degree of α-ZrP. AFM image, as seen in Figure 4c, shows that the diameter and thickness of α-ZrP platelet is 500–600 nm and 15–20 nm respectively. As depicted in Figure 4b, the exfoliated α-ZrP shows a flake-like morphology with some unique wrinkles according to the reported literatures [51,52]. The particle size distribution of ZrP nanoplates could be seen from Figure 5.

### 3.2. Characterization of Quadlayer Assembly Film

**Figure 6:**
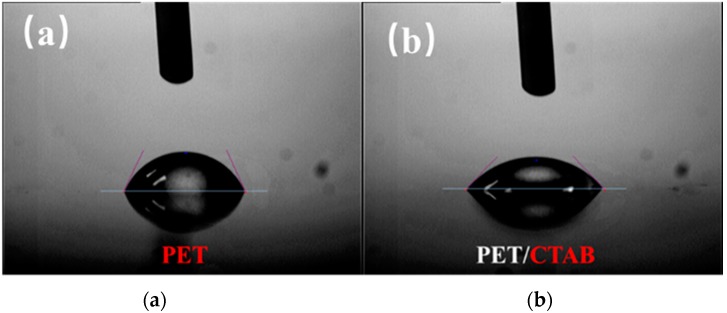
Contact angle of the surface of (**a**) neat PET and (**b**) PET/CTAB.

The hydrophobic surface of PET makes it not suitable for LbL assembly with aqueous solution even after corona treatment. In our previous work, we treated PET with 4, 4′-diphenylmethane diisocyanat (MDI), which can react with the hydroxyl groups on PET surface [41]. However, the MDI treated PET only is suitable for coating PAA solution with high concentration. Other methods, such as controlling the concentration and pH value of PAA solutions, or increasing the operation temperature, have been used to improve the wetting capability of PET surface. Here, we used CTAB as a surfactant to treat PET surface before LbL assembly. The static state contact angles were tested to evaluate the hydrophilic properties of PET films before and after CTAB treatment. As shown in Figure 6, Compared with the pristine PET, the contact angle of PET surface reduces from 68° to 45° after CTAB treatment. The lower contact angle means the hydrophilicity has been improved. As a result, a PAA aqueous solution was easily to spread uniformly over the surface of PET surface after CTAB treatment and a PAA/PDDA/ZrP/PDDA quadlayer was easily assembled onto the surface of PET.

**Figure 7:**
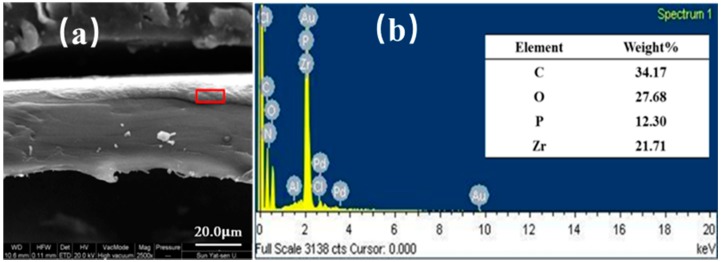
Cross sectional SEM image (**a**) and EDS result of polyethylene terephthalate quadlayer ((PET-(QL))_11_.

**Figure 8:**
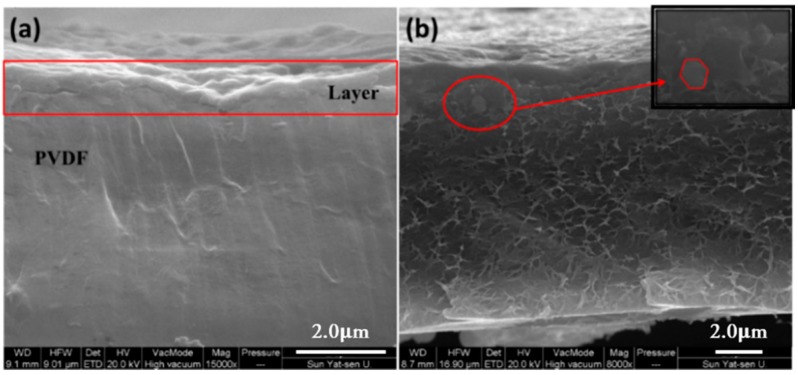
Cross sectional SEM image of 11QLs coated onto PVDF film with different fractions (**a**) and (**b**).

SEM images were used to observe the nanobrick wall structure of PET composite films. As shown in Figure 7, the borderline of PET substrate and the coating layer can be seen clearly but the thickness of the coating layer is difficult to measure because of the curling of PET substrate during sample preparation. Energy Dispersive Spectrometer (EDS) confirms the presence of α-ZrP nanoplatelets in the coating multilayer. Poly(vinylidene fluoride) PVDF-(QL)_11_ composite film was prepared for SEM examination because PVDF can be cryogenically fractured in liquid nitrogen without curling. In Figure 8, a coating multilayer structure can be observed by cross-sectional SEM image of PVDF-(QL)_11_ film, in which a tightly packed nanobrick wall architecture are formed with α-ZrP nanoplatelets as bricks and polyelectrolytes (PAA and PDDA) as mortars, as seen in Figure 1. The self-assembled multilayers are about 1 μm in the thickness of eleven quadlayers. Some scattered α-ZrP nanoplatelets from the cryogenical fracture of quadlayers shown in the cross-sectional SEM image of PVDF-(QL)_11_ film. The thickness of the composite film with 20 quadlayers is about 2 μm.

**Figure 9:**
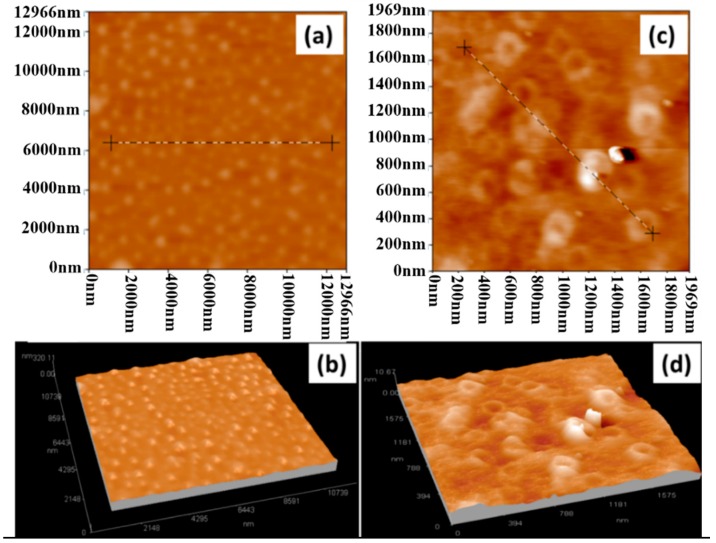
AFM Surface and 3D micrographs of silicon wafer coated with PAA (**a**,**b**) and with PAA/PDDA/ZrP (**c**,**d**).

**Figure 10:**
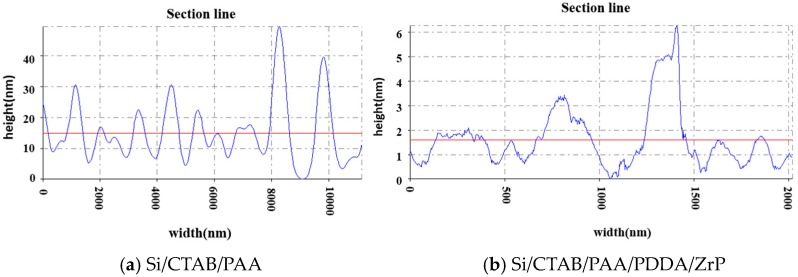
Roughness of silicon wafer coated with PAA (**a**) and with PAA/PDDA/ZrP (**b**), the solid blue line is the contour line, the solid red line is the centerline.

**Figure 11:**
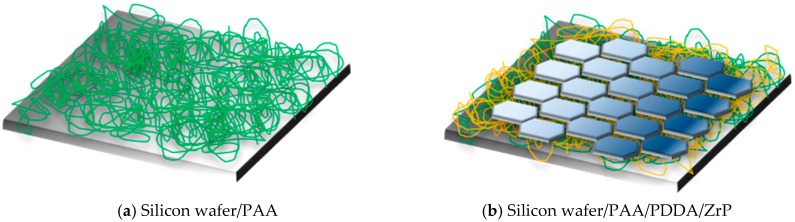
Schematics of (**a**) silicon wafer coated with PAA and (**b**) with PAA/PDDA/ZrP.

AFM was used to confirm the assembly of α-ZrP nanoplatelets with polyelectrolytes. Silicon wafer, as a hard substrate, was coated with PAA and then with PDDA and α-ZrP nanoplatelets for AFM examinations. As shown in Figure 9a,b and Figure 10a, after being coated with PAA, the surface is very coarse under the tapping mode of AFM because of the softness of polymeric coating and the maximum fluctuation is about 40 nm. After a hard layer of α-ZrP nanoplatelets is deposited on the surface by the electrostatic assembly, the surface becomes more smooth under the tapping mode of AFM, as shown in Figure 9c,d and Figure 10b. The maximum fluctuation of the surface is reduced to about 6 nm. Figure 11 gives the schematics of silicon wafer with PAA and with PAA/PDDA/ZrP on the surfaces. The decrease in roughness of the surfaces confirms that the α-ZrP nanoplatelets with negative charges were successfully assembled with the polyelectrolyte with positive charges onto the surfaces of the substrate.

**Figure 12:**
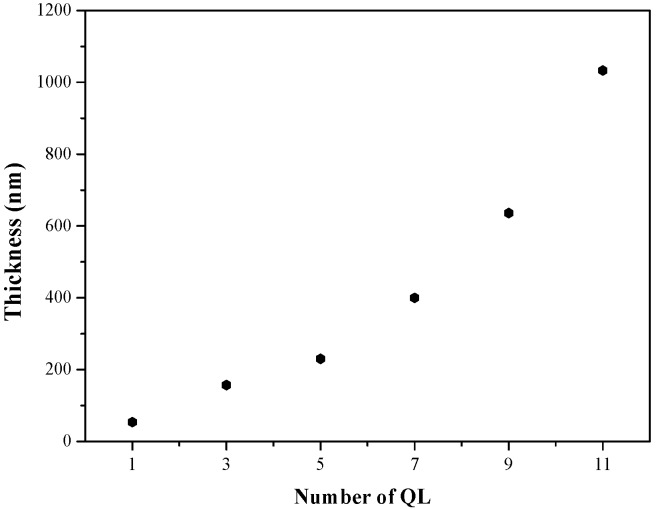
Thickness as a function of QLs deposited on PET films.

The thicknesses of QLs were also measured by the step profiler using the test method as described in the experimental section. The thickness of eleven quadlayers is 1050 nm, which is close the one measured by the cross-section SEM photograph. Figure 12 shows the thickness of the coating multilayers with different numbers of QLs. The thickness increases with the number of QLs, indicating the successfully assembly of multi-quadlayers. The thickness of each quadlayer approximates 50 nm, which is much thicker than the theoretical value. Presumably, the polyelectrolytes is not assembly as a monolayer in each QLs and the wrinkles and aggregates of α-ZrP nanoplatelets also increase the thickness of the quadlayer.

### 3.3. Gas Permeability and Transperancy

Introducing layered inorganic compounds into polymer matrix is one of the most strategies to improve gas barrier performance because the addition of layered inorganic fillers would increase the diffusion lengths of gases through the polymer matrix. The assembly of LbL multilayer has been reported to generate higher barrier films over conventional inorganic compound-filled composites, which is due to the high orientation and exfoliation of inorganic nanoplatelets in the multilayers. In this study, a 2D exfoliated α-ZrP nanoplatelets is used to fabricate LbL multilayers to improve the barrier performance of PET film. The used exfoliated α-ZrP nanoplatelet has an average with a platelet diameter of 600 nm and a thickness of approximately 1 nm. The large aspect ratio of α-ZrP nanoplatelets can induce an extremely tortuous pathway for permeating small molecules. The strong polar functional groups of polyelectrolytes also could offer superior barrier property to non-polar gases, such as oxygen gas. In this work, we combine the characteristics of polyelectrolytes and nanoplatelets to make a high oxygen barrier film.

**Figure 13:**
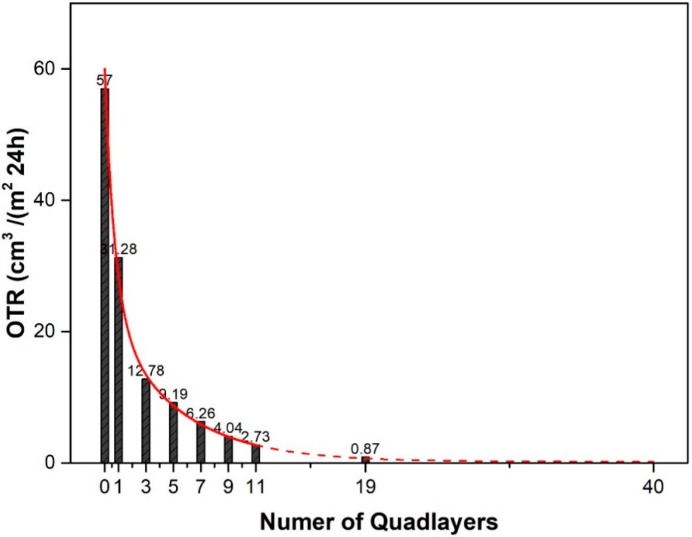
OTR values as a function of the number of QLs deposited on PET film.

The OTR and WVTR for PET-(QL)_n_ composite films were investigated to evaluate the enhancement effect of QLs on the oxygen and water vapor barrier performances of PET substrate. As shown in Table 1, we can clearly see that the OP value decreased dramatically with the increase of the QL number. Figure 13 gives the data optimization of OTR values with QL numbers (1–11QLs) for the composite film. The optimal fitting formula is as follow in Equation (5):

(3) $$\mathrm{OTR}\left(n\right)=0.33+36.67e^{-\frac{n}{0.7245}}+23e^{-\frac{n}{5.001}}$$

where n represents the number of QLs. The R-Square of this fitting formula is 0.99909.

According the fitting formula, it is found that the OTR value decreases exponentially with the increase of QL numbers. The predicted OTR value of PET composite film with 19 QLs 0.69 cm^3^/(m^2^·24 h), which is very close to the experimental data (0.87 cm^3^/(m^2^·24 h)). The OTR value of PET composite film with 19 QLs has been reduced by 98.5% of the one of the bare PET film. As a result, the fitting formula can be used to predict the OTR values of PET films with different QL numbers. The undetectable OTR value (<0.005 cm^3^/(m^2^·24 h)) is achieved by depositing 20 QLs on a PET film. The WVTR value decreases slightly with the increase of QL numbers, showing that the bare PET film has a good water vapor barrier property and the assembly of QL coating has no obvious effect on its WVTR. As we know, the hydrophilicity of the material facilitates the permeation of water vapor [1]. For example, ethylene-vinyl alcohol (EVOH) with high oxygen-barrier properties exhibits low water-vapor barrier property because of its high hydrophilicity. Here, we used polyelectrolytes (PAA and PDDA) with α-ZrP nanoplatelets to fabricate quadlayers, in which the hydrophilicity of polyelectrolytes eliminates the increasing effect of diffusivity path by 2D exfoliated nanoplatelets. These can account for the small influence of the quadlayers on water vapor barrier property.

**Figure 14:**
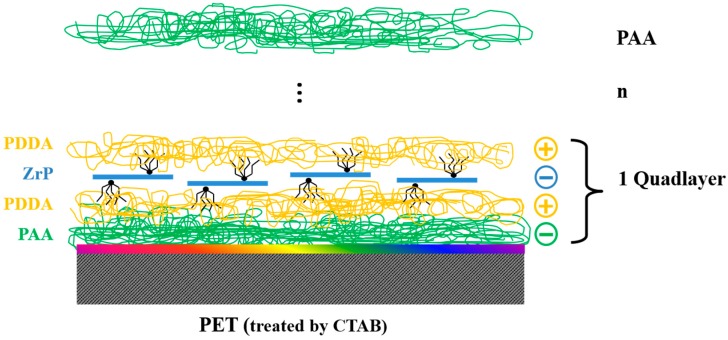
Nanobrick wall structure resulting from qualdlayer assembly of PAA (green), PDDA (orange), ZrP (blue).

The architecture of the QL assembly of α-ZrP nanoplatelets and polyelectrolytes is illustrated in Figure 14. The super oxygen barrier properties of PET-(QL)n composite films are attributed to the nano brick wall structure, which can effectively extend the diffusion length of oxygen. PET-(QL)_19_ shows a compared OP value of PVDC (reported OP is 0.01–0.03 cm^3^·mm/(m^2^·24 h)) [53], which is a commercial material with high barrier properties. PVDC is high cost and unstable during the melting process, while the composite film in this work will provide a cheap barrier film with an easy fabrication process.

**Figure 15:**
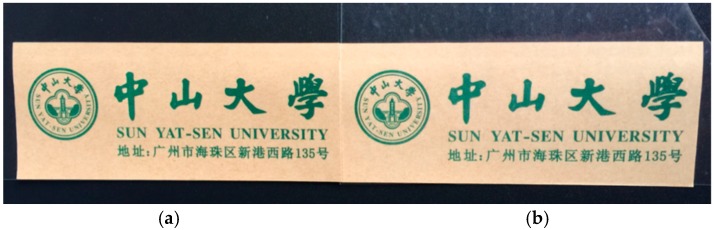
Photographs of (**a**) bare PET film and (**b**) PET-(QL)_19_ composite film in front of a printing paper.

The transparency of packing film is very important for most barrier packaging applications. As shown in Figure 15, even after depositing 19 QLs, PET composite film still has a high level of transparency, which is similar to the bare PET film. The optical transmittances and hazes of PET-(QL)_19_ and bare PET have been test and summarized in Table 2. The transparency of PET-(QL)_19_ is almost same to the bare PET film, showing the high level transparency of the QL multilayers.

## 4. Conclusions

α-ZrP nanoplatelets with a high crystalline were prepared under hydrothermal reaction condition and were well exfoliated by TBAOH. Using negatively charged α-ZrP nanoplatelets as bricks, positively charged PDDA and negatively PAA as mortars, a quadlayer coating with nanobrick wall structure were fabricated onto the surface of PET film to enhance the oxygen barrier property. The structure of QL coating was characterized and confirmed by SEM, AFM and TEM techniques. The oxygen transmission (OTR) decreased exponentially with the number of the deposited QLs. An OTR value of 0.87 cm^3^/(m^2^·24 h) was obtained by depositing 19 QLs on the surface of PET film. The corresponding OP value (0.02 cm^3^ mm/(m^2^·24 h) is reduced by 98.5% of the bare PET. It should mentioned that PET composite films still have a high level of transparency, even after deposited 19 QLs. Therefore, such QL assembly composite film shows great potential application in packaging materials.

## Figures and Tables

**Table 1:** Barrier properties of PET and PET/(QL)_n_/PAA (*n* = 0~19). OTR: oxygen transmission rate; OP: oxygen permeability coefficient; WVTR: water vapor transmission rate; WVP: water vapor permeability coefficient.

Sample	OTR (cm^3^/(m^2^·24 h))	OP (cm^3^·mm/(m^2^·24 h))	WVTR (g/(m^2^·24 h))	WVP (g mm/(m^2^·24 h))
PET	57.0	1.43	9.97	0.25
PET-(QL)_1_	31.3	0.78	9.30	0.23
PET/(QL)_3_	12.8	0.32	9.22	0.23
PET-(QL)_5_	9.19	0.23	9.15	0.23
PET-(QL)_7_	6.26	0.16	9.10	0.23
PET-(QL)_9_	4.04	0.10	9.08	0.22
PET-(QL)_11_	2.73	0.07	9.06	0.23
…	…	…	…	
PET-(QL)_19_	0.87	0.02	8.50	0.20
PET-(QL)_20_	undetectable	-	8.45	0.19

**Table 2:** Optical properties of PET and PET-(QL)_19_ films.

Sample	Transmittance (%)	Haze (%)
PET	89.5	2.0
PET/(QL)_19_/PAA	88.8	2.5
